# Phloem Exudate Protein Profiles during Drought and Recovery Reveal Abiotic Stress Responses in Tomato Vasculature

**DOI:** 10.3390/ijms21124461

**Published:** 2020-06-23

**Authors:** Aaron J. Ogden, Jishnu J. Bhatt, Heather M. Brewer, Jack Kintigh, Samwel M. Kariuki, Sairam Rudrabhatla, Joshua N. Adkins, Wayne R. Curtis

**Affiliations:** 1 Earth and Biological Sciences Directorate, Pacific Northwest National Laboratories, 902 Battelle Blvd, Richland, WA 99301, USA; Aaron.Ogden@pnnl.gov (A.J.O.); Heather.Brewer@pnnl.gov (H.M.B.); Joshua.Adkins@pnnl.gov (J.N.A.); 2 Plant Biology Graduate Program, The Pennsylvania State University, University Park, PA 16802, USA; Jishnu.Bhatt@curtislab.org; 3 Department of Chemical Engineering, The Pennsylvania State University, University Park, PA 16802, USA; Jack.Kintigh@curtislab.org (J.K.); sam.muiruri.karish@gmail.com (S.M.K.); 4 School of Science, Engineering, and Technology, The Pennsylvania State University, Harrisburg Campus, 777 W Harrisburg Pike, Middletown, PA 17057, USA; Svr11@psu.edu

**Keywords:** phloem exudate, proteomics, drought, abiotic stress

## Abstract

Drought is the leading cause of agricultural yield loss among all abiotic stresses, and the link between water deficit and phloem protein contents is relatively unexplored. Here we collected phloem exudates from *Solanum lycopersicum* leaves during periods of drought stress and recovery. Our analysis identified 2558 proteins, the most abundant of which were previously localized to the phloem. Independent of drought, enrichment analysis of the total phloem exudate protein profiles from all samples suggests that the protein content of phloem sap is complex, and includes proteins that function in chaperone systems, branched-chain amino acid synthesis, trehalose metabolism, and RNA silencing. We observed 169 proteins whose abundance changed significantly within the phloem sap, either during drought or recovery. Proteins that became significantly more abundant during drought include members of lipid metabolism, chaperone-mediated protein folding, carboxylic acid metabolism, abscisic acid signaling, cytokinin biosynthesis, and amino acid metabolism. Conversely, proteins involved in lipid signaling, sphingolipid metabolism, cell wall organization, carbohydrate metabolism, and a mitogen-activated protein kinase are decreased during drought. Our experiment has achieved an in-depth profiling of phloem sap protein contents during drought stress and recovery that supports previous findings and provides new evidence that multiple biological processes are involved in drought adaptation.

## 1. Introduction

Plant vasculature, composed primarily of xylem and phloem, is responsible for the long-distance transport of resources and molecular signals throughout the plant. The xylem functions in a passive unidirectional fashion, transporting predominately water and minerals upward from root tissue [1]. In contrast, the phloem is a bidirectional active transport system with a diverse array of molecular contents. In addition to carbohydrates produced by photosynthesis, the phloem also contains primary metabolites such as amino acids [2], secondary metabolites such as flavonoids and phenylpropanoids [3], coding and non-coding RNA [4], hormones [5], lipids [6], and protein [7,8], collectively referred to as phloem sap. Indeed, phloem contains a large and diverse collection of molecule types involved in nutrient transport, inter-organ signaling, and adaptation to stress. The phloem consists of multiple cell types, including an enucleate sieve element and a neighboring nucleate companion cell. Consequently, the sieve element is not transcriptionally active, and the mRNA, protein, and other contents of the phloem sap, therefore, reflects the activity and plasmodesmatal export from their adjoining companion cells, primarily through plasmodesmata. However, bound within the sieve elements, the phloem sap contains much of the cell’s translational machinery and may be capable of nascent protein production [7], although this has yet to be demonstrated [9]. 

To capture and study the protein profile of phloem sap, an EDTA-facilitated exudation technique is often used. The EDTA-facilitated exudation technique involves excision of the plant stem or petiole to expose sieve elements, after which the excised plant organ is placed in an EDTA-containing solution. This technique exploits the divalent cation binding capacity of EDTA to sequester Ca^2+^ ions, thereby preventing wound-induced Ca^2+^-dependent sieve element occlusion [10] and allowing the phloem sap contents to exude into a collection buffer. The EDTA, however, can cause tissue damage, cell lysis, and subsequent contamination of phloem sap exudates by the contents of neighboring non-vascular cell types. This has been partly remedied by decreasing the incubation time with the EDTA solution, followed by the collection of phloem sap exudate in distilled water [11]. While convenient, the EDTA-facilitated exudate collection technique still results in contamination by the neighboring cells [12]. For example, multiple publications identify ribulose bisphosphate carboxylase (Rubisco) and other photosynthesis-related proteins in phloem exudates, despite the apparent absence of photosynthesis in sieve elements [8,9,13]. Hence, use of EDTA even at low concentrations does likely introduce proteins that are not part of the proteome of the phloem sap, but experimental design and careful analysis can aid in its interpretation.

Reviewed by Carella et al. and Rodriguez-Celma et al., previous studies have captured and examined the protein content of phloem sap from different plant species in both stressed and stress-free conditions [14,15]. It is apparent that regardless of species, most plant phloem proteomes contain representatives of particular biological process ontologies, including redox regulation (e.g., peroxidases and oxidoreductases), protein quality control and degradation (e.g., chaperones and proteases), polysaccharide metabolism (e.g., glucosidases and galactosidases), and defense (chitinases and thaumatins) [8,15]. In response to stress, multiple reports show changes in the abundance of proteins associated with defense, lipid transport [6,16], and redox regulation [14]. 

Despite a growing understanding of the phloem sap contents in multiple species, much remains to be learned about phloem sap dynamics in response to abiotic stress. In particular, little is known about the phloem proteome of *Solanum lycopersicum* (tomato), and, to the best of our knowledge, no global protein profiling of tomato phloem sap has been performed. Tomato is both a model plant with a wealth of genetic and molecular resources [17] and an important agricultural crop, making it an attractive candidate for phloem exudate studies. Like most agricultural crops, tomato is subjected to abiotic and biotic stresses, such as drought and predation by phloem-feeding insects. Because drought results in the largest decreases in agricultural crop productivity among all biotic and abiotic stresses, the research community would benefit from an improved quantification of tomato phloem sap contents and their dynamics in response to the onset of drought stress and drought recovery.

We report here a comprehensive characterization of tomato phloem sap protein profiles during a period of drought stress and drought recovery using the EDTA-facilitated phloem exudation method followed by LC-MS/MS. Our analysis resulted in confident identification of >2500 proteins from 15 samples (5 groups of 3 biological replicates), and shows extensive consistency with previous phloem sap protein profiles. Our global dataset suggests many biological pathways beyond previous reports are present within the phloem sap. Only 31 proteins were detected uniquely in drought-stressed phloem, while the abundance of 169 proteins changed significantly during our experiment. Analysis of this subset of proteins suggests that, within phloem sap, the proteins associated with the biological processes lipid metabolism, chaperone-mediated protein folding, cytokinin biosynthesis, and branched-chain amino acid metabolism became more abundant in response to drought. Conversely, the proteins involved in particular biological processes, including carbohydrate and lipid metabolism, are became less abundant during drought stress.

## 2. Results

### 2.1. Drought and Phloem Exudate Proteomics

**Figure 1:**
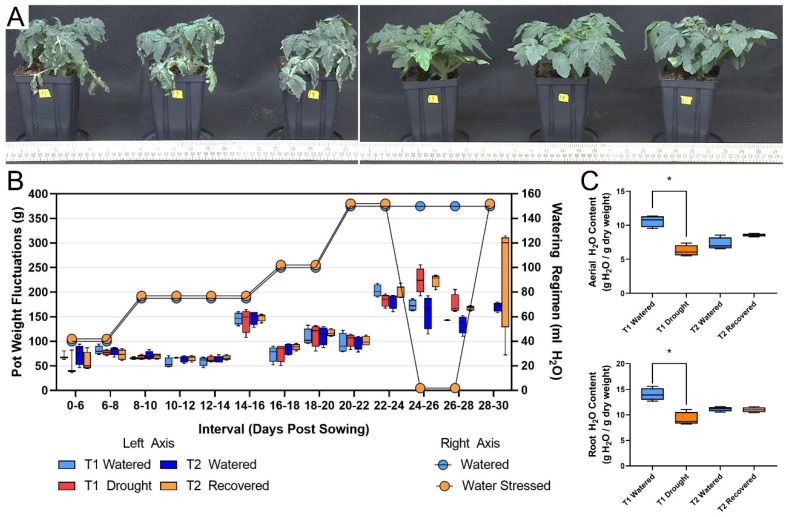
Phenotypic impacts of drought, watering regime, and impacts on plant water content. (**A**) Pictures of representative plants showing evidence of turgor pressure loss during drought at T1 (left) and after recovery at T2 (right). (**B**) Pot weight fluctuations based on total pot weights and water addition weight between regular watering intervals (left axis, box plots) and watering regimen for each watering (right axis, dots). (**C**) Water content in both aerial (top) and root (bottom) tissues during drought and recovery. Asterisks denote a t-test *p*-value < 0.05.

**Figure 2:**
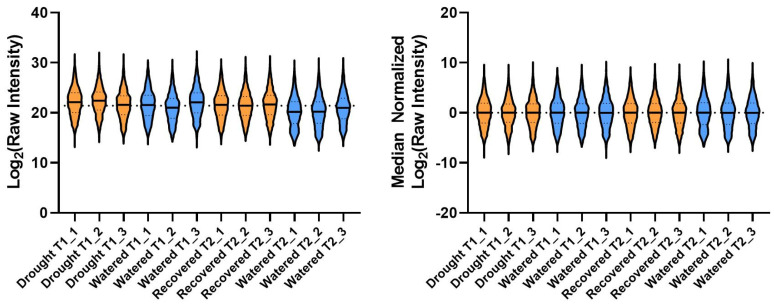
Data normalization. Violin plot representation of the raw protein abundances (**left**) and after normalization (**right**) for each sample after log_2_ transformation and median subtraction to normalize for slight differences in amounts of protein injected into the mass spectrometer. The horizontal dotted line in each graph is centered along the median protein abundance for all proteins in all samples.

To determine the impact of drought on the protein profile of tomato phloem, an experiment was executed that incorporated a period of drought stress followed by drought recovery. Phloem exudates were sampled after withholding three alternate day waterings (T1), and following drought-stressed plant recovery with ample watering over 2 days (T2). Drought-stressed tomato plants exhibited a visible loss of turgor pressure that was ameliorated upon a return to watering (Figure 1A). Throughout the experiment we observed that the fluctuations in total pot weight steadily increased, driven by plant transpiration. To prevent premature drought stress, this was accounted for by periodically increasing the watering regimen (Figure 1B). As expected, the drought-stressed plants exhibited a significant 1.5-fold reduction in both aerial and root tissue water content (g H_2_O/g tissue dry weight) (Figure 1C, Welch’s t-test *p*-value < 0.005). Phloem exudates were collected via the EDTA method for 6 h, and the protein profile of each sample was determined via LC-MS/MS. Our LC-MS/MS analysis of phloem exudate proteins resulted in the identification of 3569 proteins. To add confidence to our identifications, we reduced our dataset by limiting our analysis to proteins identified with ≥2 unique peptides, as well as to proteins that were reproducibly identified in all three biological replicates of at least one treatment group (e.g., all three drought T1 replicates). This resulted in the confident identification and quantification of 2558 *S. lycopersicum* proteins (Supplemental Table S1). Because the peptide quantities were normalized prior to injection, each dataset was median normalized to account for small differences in sample loading (Figure 2, Supplemental Table S1).

### 2.2. Tomato Phloem Exudate Proteome Confirms Previous Findings

To evaluate whether our technique successfully captured the protein profiles representative of the phloem, as well as the extent to which proteins from other cell types are present in our samples, we evaluated the identities of the most abundant proteins within our dataset. The presence of Ribulose-6 phosphate carboxylase (RbcL) in phloem exudates is sometimes used as a metric to evaluate contamination from other cell types. Although RbcL was present in our protein profile, the two most abundant proteins across all 12 biological replicates were a sieve element occlusion B-like P-protein (Solyc03g111820) and a Bet V1 domain containing protein (Solyc04g005695), each 2.5- and 2.1-fold more abundant than RbcL, respectively. Orthologues of both Solyc03g111820 [18] and Solyc04g005695 [7] have been shown to be abundant in the sieve element. A further comparison of the most abundant proteins identified across all samples from this experiment reveals a strong overlap with those identified in previous phloem exudates (Table 1, and Supplemental Table S1). For example, using NCBI protein BLAST [19], the nearest homologs of acyl-CoA binding protein (Solyc08g075690) [20], lipoxygenase C (Uniprot accession Q96573) [21], and stress-response protein (Solyc11g066950) [22] were each identified in previous phloem exudates. Similarly, the nearest homolog identified for 17 of the 20 most abundant proteins in our phloem exudate were previously identified in phloem exudate experiments [7,13,15,20,21,22,23,24,25,26,27,28,29,30]. Our analysis also identified a phloem-transported flowering time regulator, FLOWERING LOCUS T-like protein (Solyc05g053850), that was recently shown to regulate flowering time in tomato [31]. Similarly, we observe the phloem-mobile cyclophilin protein (Solyc01g111170), which was recently shown to regulate the shoot-to-root ratio, as a highly abundant protein in our phloem exudate [32]. Taken together, these data strongly support that our phloem exudate collection strategy successfully captured a protein profile representative of *S. lycopersicum* sieve elements.

### 2.3. Multiple Biological Processes are Represented in Tomato Phloem

**Figure 3:**
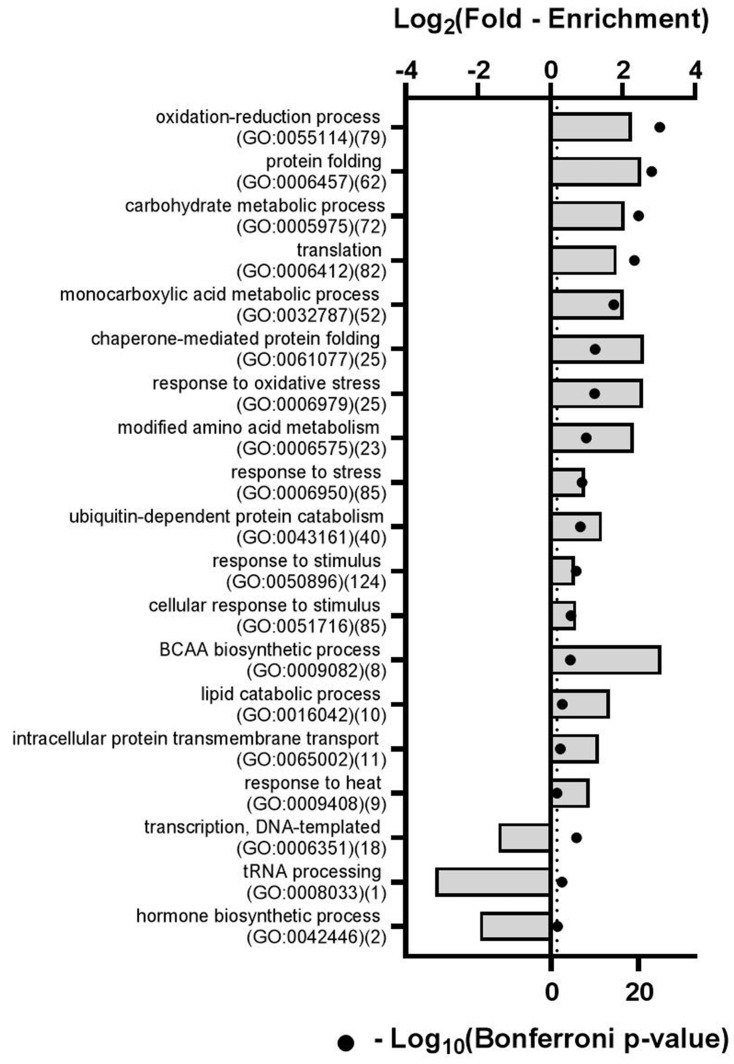
Gene ontology (GO) enrichment analysis of the total phloem protein profiles from all samples shows enrichment for multiple biological processes in the phloem. The Log_2_-fold enrichment for each GO term is represented as a bar on the top axis, and the -Log_10_ Bonferroni-adjusted *p*-values are represented as black dots on the bottom axis. The number of proteins contributing to each GO term is shown in parentheses. For reference, the vertical dotted line denotes a -Log_10_-transformed Bonferroni-adjusted *p*-value of 0.05. Additional significantly enriched GO terms can be found in Supplemental Table S2.

To determine what biological processes (BP) may occur at the protein level within the phloem we performed a gene ontology (GO) enrichment analysis on the total phloem proteome observed in all samples (i.e., all 2558 proteins from all 15 samples, Supplemental Table S1) [33]. We observed a significant over-representation of many GO:BP slim terms after a conservative Bonferroni multiple hypothesis test correction (Supplemental Table S2). Due to the redundancy in GO terms, this list was condensed using REVIGO [34], resulting in 90 reduced redundancy GO:BP terms (Supplemental Table S2, column *n*). Selected GO:BP slim terms, their Bonferroni adjusted *p*-values, and their fold-enrichment are shown in Figure 3.

Proteins belonging to multiple biological processes previously observed within the phloem were also observed in this work. For example, oxidation-reduction (GO:0055114), proteasome-mediated ubiquitin-dependent protein catabolism (GO:0043161), and translation (GO:0006412) were each significantly 4.7-, 2.7- and 3.6-fold enriched within the phloem, respectively (Bonferroni-corrected *p*-value < 0.05) [7]. Consistent with the enucleate nature of sieve elements, we observed a significant ~3-fold underrepresentation of proteins associated with transcription (GO:0042446) within the phloem exudate (Bonferroni-corrected *p*-value = 1.6 × 10^−6^) (Figure 3, Supplemental Table S2). These data suggest that the protein contents of phloem sap are complex and that multiple biological processes are likely occurring within the phloem.

### 2.4. Novel Proteins and Processes in the Tomato Phloem

The depth of our proteome facilitated the identification of proteins not previously identified in the phloem, including members of RNA silencing and trehalose metabolism. For example, members of the Argonaute protein family detected include AGO1A, AGO2A2, AGO4A, and AGO4B, (Solyc06g072300, Solyc02g069260, Solyc01g008960, and K4LP77, respectively). Expression of *ago1* in *A. thaliana* was shown to be vascular-specific [35], but to our knowledge its detection at the protein level within the phloem has not been previously reported. Similarly, three proteins predicted to be involved in trehalose metabolism were also detected in phloem exudates, including Solyc02g072150, Solyc07g055300, and Solyc08g079060. Sequence homology by BLASTp suggests that Solyc02g072150 and Solyc07g055300 are homologous to *A. thaliana* trehalose-phosphate synthases, while Solyc08g079060 is homologous to an *A. thaliana* trehalose-phosphate phosphatase. Our analysis also identified eight proteins involved in branched-chain amino acid metabolism (BCAA, GO:0009082), and three proteins involved in K-63 linked protein ubiquitination (GO:0070534) (Supplemental Table S2). Similarly, 15 and 25 proteins associated with reactive oxygen species (ROS) metabolism and chaperone-mediated protein folding, respectively, were identified in the phloem. However, six of the 15 proteins associated with ROS metabolism were identified in previous phloem exudate studies (Supplemental Table S2). These data suggest that RNA-silencing, trehalose metabolism, BCAA biosynthesis, and other previously unreported biological processes are present within the phloem.

### 2.5. Drought Stress Alters Phloem Proteome

To determine the extent to which water deprivation impacts the phloem protein profiles, we performed a drought experiment followed by protein profiling of the phloem exudates (Figure 1). Drought stress, particularly the loss of turgor pressure, was apparent at Timepoint 1 (T1) after withholding three alternate-day waterings (Figure 1A). As shown by a clear increase in turgor pressure and leaf tissue water content, drought symptoms were alleviated at Timepoint 2 (T2), two days after a return to watering (Figure 1A). Only 31 proteins were identified exclusively in phloem exudates of the drought-stressed plants (Supplemental Table S1). While no particular protein ontology was enriched within this subset of unique drought-specific proteins, members include two lipid transfer proteins (Solyc01g081600, and UniProt accession O24024), a universal stress protein (Solyc04g014600), and 12 uncharacterized proteins.

**Figure 4:**
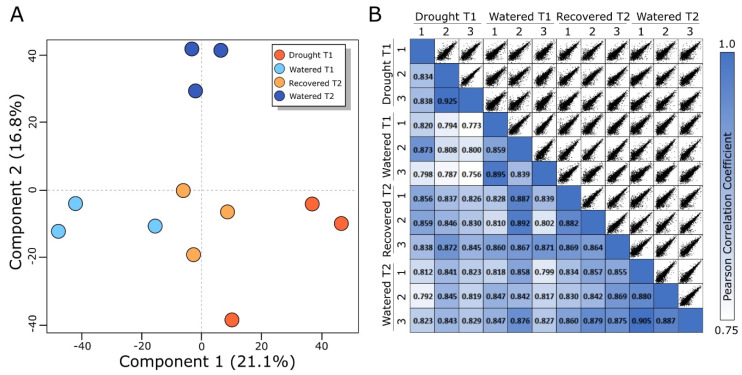
Statistical analysis of each treatment group. (**A**) Principal component analysis score plot of each sample colored by treatment, showing separation of each treatment group. (**B**) A pairwise Pearson correlation matrix of each sample showing the individual correlations between all samples.

To evaluate whether changes in protein abundances collected from phloem exudates were statistically distinguishable, we performed a principal component analysis (PCA) (Figure 4A). Component 1 of the PCA score plot comprises 21.1% of the data variation and is caused by changes between the T1 watered and T1 drought-stressed plants. The second component, comprising 16% of the data variation, is caused by differences between the T2 watered and all other samples, suggesting the phloem protein profiles change over time as plant size increases for these growth conditions. The location of the T2 drought-recovered plant samples on the score plot is between the T1 watered and T1 drought samples, consistent with a partial return to pre-drought levels. A pairwise comparison and Pearson correlation coefficient (PCC) was calculated using the abundances of each protein profile for all samples (Figure 4B). The PCCs ranged from 0.755 to 0.925, suggesting a relatively strong positive correlation between the phloem exudate protein profiles from all samples, regardless of drought. However, the lowest correlations observed were between the T1 drought and T1 watered samples (PCC range from 0.756 to 0.873). These PCA and PCC data suggest that drought impacts the phloem sap protein content and that partial restoration of pre-drought protein abundances occurs after plants are returned to the healthy watering regimen.

**Figure 5:**
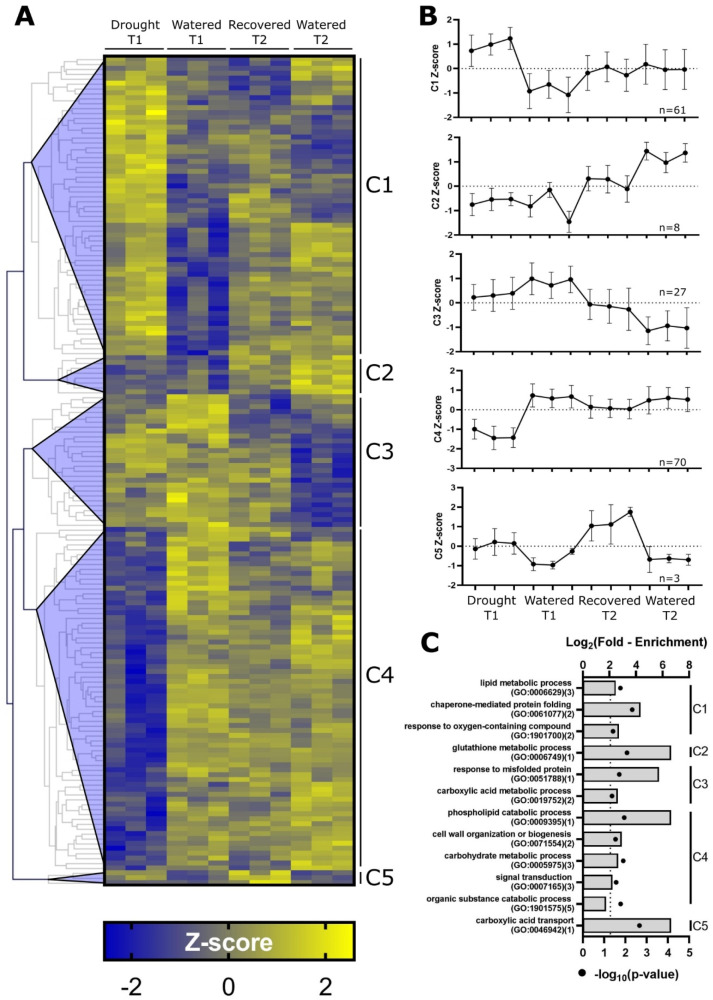
Hierarchical cluster analysis (HCA), cluster-specific Z-score trends, and gene ontology (GO) enrichment analysis of each cluster. (**A**) Hierarchical clustering analysis of 169 proteins with an adjusted ANOVA *p*-value <0.05. (**B**) Z-scores from each cluster averaged by sample. Error bars denote standard deviation. The number of proteins in each cluster is shown on each graph. (**C**) Selected ontology terms enriched among proteins from each cluster (C1–C5) via the Panther overrepresentation test (Bonferroni-adjusted *p*-value < 0.05). The Log_2_-fold enrichment for each GO term is represented as a bar on the top axis, and the associated -Log_10_-adjusted *p*-values for each GO term are represented as black dots on the bottom x-axis. The number of proteins contributing to each GO term is shown beside the respective term in parentheses. For reference, the vertical dotted line denotes a -log_10_-adjusted *p*-value of 0.05. Additional GO terms can be found in Supplemental Tables S3–S7. T1, Timepoint 1; T2, Timepoint 2.

Of the 2558 proteins confidently identified in our study, the abundances of 169 proteins were significantly changed between the different samples (ANOVA *p*-value < 0.01, 10% FDR, Supplemental Table S1). To further understand the regulation of these 169 proteins, we performed a hierarchical clustering analysis (Figure 5A, Supplemental Table S1), which resulted in five distinct clusters (C1–C5, Figure 5B). Cluster C1 contains the proteins whose abundances increase in response to drought, while the abundances of proteins in Cluster C4 decrease during drought. Cluster C2 appears to contain proteins whose abundances increase naturally during plant development from T1 to T2 under non-drought conditions but are suppressed in response to drought. Similarly, phloem proteins in Cluster C3 appear to decrease from T1 to T2 when plants are well watered but remain at an intermediate elevation in T2 recovered samples. Interestingly, Cluster C5 contains proteins that are mildly elevated in response to drought at T1 and increase substantially in abundance after a return to watering in T2 drought-recovered samples.

### 2.6. Positively Regulated Drought-Responsive Proteins

To identify the proteins whose abundance changed during our experiment, a permutation-based FDR-adjusted ANOVA was performed and only proteins with an adjusted *p*-value threshold < 0.01 are included in this section. To determine whether a protein abundance changed specifically in response to drought, further analysis of T1 drought and T1 watered was performed by a post-hoc t-test using only the 169 proteins with a significant change as determined by the ANOVA. Of particular interest are the 61 increased drought-responsive proteins belonging to Cluster C1 (Figure 5B). The enrichment analysis revealed that among the C1 proteins multiple GO terms, including the lipid metabolic process (GO:0006629), chaperone-mediated protein folding (GO:0061077), as well as response to oxygen-containing compound (GO:1901700), are significantly 5.6-, 20.3-, and 6.6-fold overrepresented, respectively (Figure 5C, Supplemental Table S3) (Bonferroni-adjusted *p*-value ≤ 0.02). Proteins within the lipid metabolism GO term include the temperature-induced lipocalin (TIL) (Solyc07g005210), which showed a significant 48-fold increase in response to drought (post-hoc t-test *p* = 0.02). Members of the chaperone-mediated protein folding term (GO:0061077) in Cluster C1 include the likely HSP70 protein (Solyc10g086410) and likely GROES-like protein (Solyc07g008800), both of which exhibiting a significant 1.6- and 2-fold increase in response to drought, respectively (post-hoc t-test *p* = 0.04 and *p* = 0.001, respectively). The abscisic acid and environmental stress-inducible dehydrin protein TAS14 (P22240, Solyc02g084850) is a member of the “response to oxygen-containing compound” GO term family and exhibited the largest drought-induced increase in protein abundance, reaching a 238-fold higher abundance during drought (post-hoc t-test *p* = 0.03).

Independent of the ontology overrepresentation tests, members of the 61 Cluster C1 proteins suggest multiple processes are impacted by drought within the phloem, including amino acid and carboxylic acid metabolism, abscisic acid signaling, and lipid metabolism. For example, the Δ^1^-pyrroline-5-carboxylate synthase (P5CS, Solyc06g019170), involved in proline biosynthesis and plant osmoregulation [36], is a member of Cluster C1 and significantly 1.5-fold more abundant in response to drought (post-hoc t-test *p* = 0.002). Similarly, the Bet v1 domain-containing protein (Solyc04g007820), likely involved in fatty acid and/or cytokinin binding [37], is significantly 2.6-fold increased during drought (post-hoc t-test *p* = 0.04). We also observed a significant 8-fold increase in the abundance of the cytokinin activating protein (Solyc08g062820) during drought (post-hoc t-test *p* = 0.01). The semi-aldehyde dehydrogenase domain-containing protein (Solyc01g005250), also in Cluster C1 (significantly 2-fold increased by drought, post-hoc t-test *p* = 0.01), is likely involved in the metabolism of amino acids, including isoleucine, lysine, and methionine. Two additional lipid-related proteins, the plastid-lipid-associated protein O24024 and non-specific lipid-transfer protein Solyc01g081600, had respectively 5.5- and 4.8-fold increase in response to drought (post-hoc t-test *p* = 0.002 and 0.0004, respectively). These observations suggest that tomato responds to drought by altering its amino acid metabolism, stress response, cytokinin, and carbohydrate metabolism within the phloem.

### 2.7. Downregulated Drought-Responsive Proteins

Cluster C4 is comprised of proteins whose abundance in the phloem exudate apparently decreases in response to drought. The Panther enrichment analysis revealed a significant >100-, 7.7-, 6.3-, 4.7-, and 3.4-fold overrepresentation of proteins belonging to the GO terms phospholipid catabolic process (GO:0009395), cell wall organization or biogenesis (GO:0071554), carbohydrate metabolic process (GO:0005975), signal transduction (GO:0007165), and organic substance catabolic process (GO:1901575), respectively (Figure 5C, Supplemental Table S4). The only protein within the phospholipid catabolism GO category is a likely non-specific phospholipase (Solyc01g008790), whose abundance was significantly 17-fold reduced in response to drought (post-hoc t-test *p* = 0.002). While members of the signal transduction GO category significantly changed by ANOVA, including the xyloglucan endotransglucosylase (Solyc03g093130), 12-oxophytodienoate reductase (Solyc10g086220), and the likely HIPL1 protein (Solyc03g113470), only the endotransglucosylase significantly decreased 34-fold during drought (T1 watered vs. T1 drought post-hoc t-test *p* = 0.01).

Independent of the overrepresentation tests, additional members within Cluster C4 include proteins likely involved in carbohydrate metabolism and primary cell wall remodeling. For example, the abundance of α-L-arabinofuranosidase (Solyc12g100120), O-glycosyl hydrolases (Solyc05g050130 and Solyc01g104950), and the xyloglucan endotransglucosylase (Solyc03g093130) were each significantly 3.7-, 78-, 9.2-, and 34-fold reduced during drought, respectively (post-hoc t-test *p* < 0.05). Two of the three proteins with the largest reduction in abundance during drought are glycoside hydrolases Solyc04g077190 and Solyc05g050130, which show a 111- and 78-fold decrease (post-hoc t-test *p* < 0.009). Similarly, the likely fasciclin-like arabinogalactan Solyc07g045440 was significantly 6-fold reduced during drought (post-hoc t-test *p* = 0.003). These observations suggest that aspects of carbohydrate metabolism within the phloem sap may be altered in response to drought.

Other proteins with large reductions in abundance during drought include likely A1-domain-containing peptidases Solyc06g068550, Solyc03g117690, and Solyc08g067100, each of which are significantly 168-, 51-, and 14-fold less abundant, respectively (post-hoc t-test *p* < 0.006). We also observed a significant 4.3-fold reduction in the abundance of the mitogen-activated protein kinase 3 (MAPK3, Solyc06g005170) (post-hoc t-test *p* = 0.02). The neutral ceramidase protein Solyc03g006140, likely involved in sphingosine metabolism, was 2-fold decreased in abundance during drought (post-hoc t-test *p* = 0.03). Taken together, these data suggest that particular aspects of peptidase metabolism, lipid metabolism, and at least one likely MAP kinase is decreased in tomato phloem during drought.

### 2.8. Persistent Post-Recovery Impact of Drought on Phloem Protein Profiles

During drought, the abundance of proteins in clusters C1 and C4 apparently increase and are decreased, respectively, before returning to the approximate abundance observed in watered T2 plants, suggesting that these proteins return to non-drought stressed levels upon recovery from drought stress. However, of the 169 proteins that changed significantly via ANOVA, 40 proteins remained significantly changed between T2 drought recovered and T2 watered samples (post-hoc t-test, 5% FDR, supplemental Table S1). From clusters C1 (*n* = 61) and C4 (*n* = 70), 16 and 11 proteins remain elevated and suppressed, respectively, upon a return to ample watering and relief of drought at T2. The remaining 13 proteins that do not appear to return to non-drought homeostatic levels belong to Clusters C2 (*n* = 2), C3 (*n* = 10), and C5 (*n* = 1) (Figure 5B). In Cluster C2, for example, a thioredoxin domain-containing protein (Solyc02g068500) and a likely glutathione hydrolase (Solyc12g008640) are significantly 2.1- and 5.3- fold less abundant in the T2 recovered versus T2 watered samples, respectively (post-hoc t-test *p* < 0.02). Conversely, members of the Cluster C3 proteins include a lipase domain-containing protein (Solyc01g100930) and an uncharacterized but likely ubiquitin-fusion degradation protein (Solyc01g110410), both of which remained significantly 9.4- and 5.6-fold more abundant in recovered T2 compared to watered T2. Of the three proteins in Cluster C5, only the pyruvate carrier protein (Solyc08g082760) remained significantly 48-fold more abundant after drought recovery (recovered T2) compared to watered T2 plants (post-hoc t-test *p* = 0.0003). These data suggest that while most drought-responsive proteins quickly return to pre-drought abundance levels, some processes remain elevated or suppressed. Members of these clusters, as well as the ANOVA and post-hoc analyses can be found in Supplemental Table S1.

## 3. Discussion

The phloem is a vital plant system necessary for the distribution of photosynthates, hormones, and many other signaling molecules between distant plant parts. Understanding the contents of phloem sap and their change in response to different stimuli is therefore crucial to improve crop performance [38]. To this end, our study employed the EDTA-facilitated method to capture the phloem exudates of tomato leaves as they endure and recover from drought. As indicated in prior reports, the EDTA method has technical limitations, but nevertheless has successfully identified phloem sap contents that are subsequently confirmed by orthogonal techniques. Our analysis did identify Rubisco (RbcL), indicating partial contamination of the non-vascular photosynthetic cells surrounding the phloem, and likely xylem sap. However, a careful comparison of the most abundant proteins in our dataset with previous findings indicate successful enrichment for phloem-specific proteins.

Our proteomics workflow consisted of nano-UPLC peptide separation followed by ESI-MS/MS and resulted in the confident identification of >2500 proteins. The use of gene ontology enrichment (GO) analyses is a commonly employed strategy to determine whether particular biological pathways are overrepresented within a dataset more so than would be expected by chance given the natural abundance of those proteins within the genome. Phloem remains a relatively understudied tissue, however, and consequently few GO terms are associated with phloem processes. Nevertheless, we applied GO enrichment analyses to both our total phloem protein profile, as well as those proteins whose abundance are significantly changed by drought. Our GO enrichment analysis of the total phloem profile resulted in a surprisingly large number of significantly enriched GO terms after a conservative Bonferroni *p*-value adjustment [33]. Conversely, our GO enrichment analysis also found a significant underrepresentation of transcription-related proteins, consistent with the enucleate nature of sieve elements. Examples of previously identified phloem proteins identified in our exudate include the flowering locus T-like protein (Solyc05g053850), the sieve element occlusion *p*-protein (Solyc03g111820), and the BET v1 protein (Solyc04g005695).

While our total exudate protein profile is enriched for processes previously identified within the phloem, including proteasome-mediated protein catabolism and ribosomal systems [39], we also identified novel biological processes in the phloem. For example, we observed a large number of proteins involved in chaperone-mediated protein folding within the phloem. Chaperones have been identified in multiple previous phloem exudate studies and were shown to be suppressed in the phloem of *A. thaliana* during infection with the pathogen *Pseudomonas syringae* [8]. However, the large number of chaperones identified here suggests that independent of drought, much more of the phloem protein landscape is dedicated to protein trafficking and quality control than previously thought. Similarly, a large number of branched-chain amino acid (BCAA) biosynthetic proteins were detected in the total phloem. While BCAAs comprise only a relatively small portion of the free amino-acid pools of tomato phloem, their abundance is increased during infection with tomato yellow leaf curl-virus [40]. Amino-acid loading to and from the phloem is well studied, and our findings suggest BCAA synthesis may also occur within the phloem and become enhanced during drought stress to provide energy compensation during reduced photosynthate production [38,41]. Our total phloem profile is also enriched for three K63 ubiquitinating E2 ligase proteins. In addition to potentially targeting proteins for degradation by the proteasome, K63 ubiquitin modifications may regulate protein activity and localization. Phloem proteins are known to be K63 ubiquitinated [39], and our findings suggest three candidates as E2 ligase enzymes responsible for targeting particular phloem proteins for ubiquitination (Supplemental Table S1). Our experiment also identified multiple members of the Argonaute protein family, which play a central role in RNA silencing. Previous reports indicate Argonaute gene expression in *A. thaliana* vasculature [35,42] and suggest possible roles in anti-viral gene silencing. Our data indicate small RNA processing systems are indeed present in the phloem, including AGO1. It should be noted, however, that without further experimentation, we are unable to say conclusively that these proteins are actively functioning within the phloem.

In addition to identifying new proteins and processes in the total phloem sap protein profile as a whole, our experiment also sought to identify the drought-responsive proteins by withholding watering from a subset of test plants. Our comparative analysis consisted first of an ANOVA, which resulted in 169 proteins with an adjusted *p*-value threshold < 0.01. To determine whether these 169 proteins changed specifically in response to drought, we also performed post-hoc t-tests using drought T1 and watered T1 samples, resulting in 127/169 proteins with a significant change, indicating many of the changes observed by ANOVA are indeed caused by drought. Among these 127 proteins are many known and novel drought-responsive proteins. For example, the dehydrin protein TAS14 (Solyc02g084850) has been shown to be expressed in phloem companion cells and to become transcriptionally upregulated in response to abscisic acid, elevated salt, and other stresses [43]. Our experiment identified TAS14 and found its abundance in the phloem to increase significantly in response to drought, likely to protect proteins or membranes from damage [44]. Similarly, we show a significant increase in the temperature-induced lipocalin (TIL) during drought. TIL is known to be necessary for thermotolerance in *A. thaliana* [45] and may interact with sucrose transporters in potato [46]. The drought-increased Bet v1 protein we identified has also been detected in previous phloem exudates [25,47] and likely binds to lipids [6,11], as well as fatty acids and cytokinins [37], but to the best of our knowledge has not been previously associated with drought stress.

We observed a significant increase in the phloem abundance of Δ^1^-pyrroline-5-carboxylate synthase during drought (P5CS, Solyc06g019170). The P5CS protein was originally shown to be involved in proline biosynthesis and inducible upon salt-shock in drought-resistant moth bean [36]. Overexpression of P5CS has since been shown to confer osmotic stress tolerance in tobacco [48], potato [49], and rice [50]. Our data, therefore, suggests that tomato may similarly adapt to drought by upregulating P5CS and increasing the levels of osmoprotectants, such as proline. Our observation that the cytokinin biosynthesis protein Solyc08g062820 is significantly more abundant in the phloem during drought stress is consistent with previous observations in maize [51]. Cytokinin is a plant hormone involved in many biological processes, and cytokinin levels in phloem sap have been linked to flowering time [52,53,54]. Comprehensively reviewed by Paul et al. [55], while cytokinin levels appear to decrease in vascular tissues of poplar during drought, they also point out that elevating the cytokinin biosynthesis genes under a stress-inducible promoter confers enhanced drought tolerance in multiple species [56,57,58,59]. Our observation that a cytokinin biosynthetic protein is induced upon drought stress suggests cytokinin biosynthesis in tomato phloem may play a similar role in adaptation to drought stress.

The semi-aldehyde dehydrogenase domain-containing protein Solyc01g005250 is also significantly more abundant upon drought stress. Aldehyde dehydrogenases are a large protein family likely involved in the oxidation of aldehydes to carboxylic acids and are known to be both up and downregulated during drought stress in soybean [60]. Overexpression of some aldehyde dehydrogenases was shown to confer oxidative and osmotic stress tolerance in *A. thaliana* [61]. It is possible that the increased abundance of our observed semialdehyde dehydrogenase functions to prevent accumulation of aldehydes formed during oxidative stress. Another phloem protein that substantially increased during drought is the non-specific lipid-transfer protein (nsLTP) Solyc01g081600. The nsLTP proteins have been shown to bind phospholipids, glycolipids, steroids, and acyl-CoAs [62], as well as confer additional drought [63] and pathogen [64] stress tolerance. Lipid transfer proteins have been implicated in long-distance systemic signaling in plants, and Solyc01g081600 may function similarly within the phloem during drought by maintaining membrane homeostasis or long-distance lipid transport.

Multiple proteins were also detected whose abundance decreased significantly during drought. For example, we observed a significant reduction in a Calreticulin protein (CRT), which is known to participate in the endoplasmic reticulum as a Ca^2+^-binding protein chaperone, and also localize to the plasmodesmata in maize [65,66]. Interestingly, CRT is induced upon drought in *G. max* and results in added drought tolerance when expressed in tobacco, but is significantly reduced upon drought in *Quercus robur* [67]. It has been suggested that CRT may play a role in determining the strength of the source-sink relationships in the phloem by controlling phloem unloading and are likely to be different in annual versus perennial plants [68,69]. We also observe a significant reduction in a likely fasciclin-like arabinogalactan (FLA) protein. The FLA protein was previously identified in phloem exudates [8] and also shown to be downregulated in response to a pathogen challenge [70] and drought stress [71]. The FLA protein family is involved in plant growth, development, biotic and abiotic stress adaptation, and are expressed within vascular tissues [71,72]. The observed tomato FLA is most homologous to *A. thaliana* FLA2 (At4G14360), which was shown to rapidly decrease at the transcript level in response to abscisic acid and may play a role in shoot tissue regeneration [73]. It is likely that the FLA2 decrease in phloem sap functions similarly in signaling that leads to changes in stomatal behavior and root morphology [73].

We also observed a significant reduction in a neutral ceramidase Solyc03g006140 protein during drought. The neutral ceramidase proteins are involved in sphingolipid metabolism by hydrolyzing ceramide to sphingosine and a fatty acid. Our ceramidase Solyc03g006140 is most homologous to the *Arabidopsis* neutral ceramidase AtNCER1 (At1g07380), which was shown by knockout to accumulate hydroxyceramides and become sensitive to oxidative stress [74]. While few studies exploring hydroxyceramide accumulation in plants exist, many reports show a link between hydroxyceramide levels and cell signaling, membrane homeostasis, and cell death in mammals [75]. However, it was recently shown that an *Arabidopsis Atncer1* knockout resulted in elevated hydroxyceramide levels, accumulation of jasmonoyl-isoleucine in leaves, and resulted in early leaf senescence [76]. Our findings suggest that ceramide metabolism in the phloem might prepare plants for leaf loss as a proactive mechanism for severe drought.

We observed a large decrease in abundance of an O-glycosyl hydrolase (GH) domain-containing protein Solyc05g050130. The GH protein family hydrolyzes carbohydrates from other molecules, and have been linked to many biological processes [77] but are most likely involved in cell wall modification [78,79]. Another substantially decreased phloem sap protein, the xyloglucan endotransglucosylase (XTH, Solyc03g093130), is also likely involved in cell wall development, and similar XTH proteins are known to be involved in vascular cell wall expansion in poplar [80]. Our data suggest that particular aspects of cell wall remodeling may be decreased during drought stress, although the lack of experimental validation of the GH and XTH protein function precludes us from suggesting a specific mechanism. Interestingly, we also observed a significant decrease in a mitogen-activated protein kinase protein Solyc01g094960. Previous reports have linked MAPK3 proteins to virus tolerance [81], botrytis tolerance [82], and drought tolerance in *Solanum pimpinellifolium* [83]. To date, the phosphorylation status of phloem sap proteins has not been explored, and our findings suggest that a changing phosphoproteome within the phloem may have a role in adaptation to drought stress.

Our study has identified a large number of known and novel proteins within phloem sap, as well as proteins whose abundance changes in response to drought. Novel phloem sap proteins identified here include those associated with ROS, BCAA, and trehalose metabolism, as well as RNA silencing. Drought-responsive proteins include those associated with thermotolerance and osmoprotectant production, lipid metabolism, cell wall modification, ceramide metabolism, and mitogen-activated protein phosphorylation. While further experiments are necessary to confirm the activity of such proteins within the phloem sap, these findings open multiple avenues for future research of the role of plant vasculature in drought-stress adaptation.

## 4. Materials and Methods

### 4.1. Plant Growth

*Solanum lycopersicum* cv. “Florida Lanai” seeds were collected from plants grown in greenhouse conditions. All subsequent growth was carried out in a Percival growth chamber (AR75L3X configured with white LED tubes and red/far-red LED light bars) with light levels set at 50 and 100% for red and white light, respectively, resulting in ~100 W/m^2^ (~400 PAR) light as quantified with a LI-COR light logger (LI-COR Biosciences, Li-1500, Lincoln, NE, USA) equipped with a LI-COR pyranometer sensor (Li-200R, Lincoln, NE, USA). To begin the experiment, two seeds were sown into 4.12” × 5” (Dillen Products, DSQVP45PF/D, Middlefield, OH, USA) containing 300 g of Scotts Miracle Gro™ soil (Scotts, Marysville, OH, USA) moistened with 200 mL of water prior to sowing. Pots were covered with plastic wrap and not watered for 6 days during germination. Upon germination, 6 days post sowing (DPS), the plants were uncovered and thinned to a single plant per pot. The initial watering regimen consisted of 40 mL H_2_O every other day starting at 7 DPS. The growth chamber conditions facilitated rapid growth and transpiration, necessitating an increase in watering to prevent premature drought stress, noted in Figure 1. At 25 DPS, 6 biological replicates of plants selected at random were subjected to drought stress by withholding water for an additional 4 days, while control plants continued to receive 150 mL H_2_O every other day. After skipping 3 alternate day waterings (totaling 6 days since the last watering), phloem exudate was captured from half of the drought-stressed plants at 29 DPS as the initial comparative drought condition, constituting Timepoint 1 (T1). Drought-recovered plants were watered 150 mL H_2_O on 29 and 30 DPS. A second phloem exudate was collected from both the drought-recovered and watered plants, constituting Timepoint 2 (T2), at 31 DPS. Tissue dry weights at harvest were obtained by collection of the entire (minus sampling) aerial or root mass (obtained with thorough soil removal by rinsing) and placement in pre-tared foil pouches dried at 70 °C for 3+ days, followed by closing of the foil pouches; an additional day of drying followed by cooling under desiccation before final weights. Weights were repeated after subsequent further drying to confirm a constant dry weight (DW).

### 4.2. Phloem Exudate Collection

Phloem exudate was collected as previously described with minor modifications [84]. Briefly, leaf three was excised at the petiole from three biological replicate tomato plants (each at the 5/6 leaf-stage) using a scalpel. The cut petiole of each excised leaf was transferred immediately into a solution containing 20 mM EDTA for 30 min in a humid chamber followed by immersion in distilled water with 0.4 mM Roche Pefabloc SC Plus (Sigma-Aldrich cat#11873601001, St. Louis, MO, USA) to prevent protein degradation within the captured exudate. Phloem exudates were collected inside a humid chamber for six hours in the dark, at which point the phloem exudates were snap-frozen in liquid nitrogen.

### 4.3. Protein Sample Preparation and LC-MS/MS

Frozen phloem exudate was cryogenically lyophilized and resuspended in 8 M urea in 50 mM NH_4_HCO_3_. Protein was then quantified via Pierce BCA (Thermo Scientific, Cat# 23225, Waltham, MA, USA). Protein disulfide bonds were reduced by the addition of dithiothreitol to a final concentration of 20 mM and incubation at 37 °C for 1 h with shaking at 800 rpm. Alkylation was achieved by the addition of iodoacetamide to a final concentration of 40 mM with continued shaking at room temperature. Prior to digestion, each sample was diluted 8-fold with 1.14 mM CaCl_2_ in 50 mM NH_4_CO_3_, bringing the final CaCl_2_ concentration to 1 mM. Protein was then quantified via Pierce BCA (Thermo Scientific, Cat# 23225, Waltham, MA, USA). Trypsin was added to each sample at a 1:50 w:w trypsin-to-protein ratio followed by incubation at 37 °C for 3 h with shaking at 800 rpm. Each sample was then cleaned with C-18 solid-phase extraction columns (Phenomenex, Cat# 8B-S001-DAK, Torrance, CA, USA), and the resulting peptides were quantified by BCA. A total of 5 μL of 0.1 μg/μL peptide was injected into Waters nanoAcquity liquid chromatography system and separated using a reverse-phase C-18 column (in-house prepared 70 cm × 70 μm i.d., 3 μm Jupiter C-18) in-line with a Q-Exactive Plus mass spectrometer (Thermo Fisher, Waltham, MA, USA) at 300 nL/min. Separation occurred over 100 min using a linear gradient of 0.1% formic acid in water (A) to 0.1% formic acid in acetonitrile (B). Eluent entered the mass spectrometer via electrospray ionization in the positive mode.

### 4.4. Data Analysis

The RAW files for each LC-MS/MS analysis can be accessed via ProtomeXchange with the dataset identifier PXD018993 [85,86]. For all samples, a peptide search was performed via MaxQuant (V1.6.5.0) [87] Andromeda [88] using a comprehensive FASTA generated from the UniProtKB [89] database entries (Both Swiss-Prot and TrEMBL) for *Solanum lycopersicum* (Taxon identifier 4081, SOLLC). The Maxquant search parameters included variable modifications of methionine oxidation and N-terminal protein acetylation, as well as fixed modifications of carbamidomethylation (maximum of 5 modifications per peptide) using the re-quantify function. Global parameters also included a “match between run” window of 1.4 min, a 1% protein false discovery rate, and protein abundance estimation via iBAQ. The resulting combined protein groups file was then imported into Perseus [90], where contaminants were removed from the dataset. To add confidence to our protein identifications, the dataset was further reduced by filtering for proteins that were identified using ≥2 unique peptides. Furthermore, only proteins that were detected in all three biological replicates of at least 1 treatment group (e.g., all replicates from drought T1 or watered T1, etc.) were considered for subsequent analyses. The resulting 2558 proteins were then log_2_-transformed, followed by missing value imputation from a normal distribution with a width of 0.3 and a downshift of 1.8 [90]. Normalization was achieved by a median subtraction for each sample (Figure 2). An ANOVA was performed with a permutation-based FDR of 10% using 250 randomizations. For hierarchical clustering analysis (HCA) and heatmap generation, proteins with an adjusted ANOVA *p*-value threshold < 0.01 were Z-score transformed (z = χ − μ/σ) [90,91] and imported into Multiple Experiment Viewer MeV (v4.9.0). The HCA was performed using average linkage and Euclidean distances [92]. Cluster number was determined using the MeV Figure of Merit function [93]. An additional post-hoc t-test with permutation-based 5% FDR correction was performed using the drought T1 versus watered T1 samples, and recovered T2 versus watered T2 samples. Each identified protein, their abundance, and relevant statistics can be found in Supplemental Table S1. When used, gene ontology enrichment analysis was performed using the Panther [33] (v.15.0, Annotation version 15.0, Released 14-Feb-2020) GO-Slim biological process terms. Panther settings for enrichment analysis of the entire phloem protein profile (Supplemental Table S2), as well as the individual clusters (Supplemental Tables S3–S7), consisted of a Fisher test-type and a Bonferroni multiple test correction using the entire tomato genome as a background. Results of the Panther enrichment tests can be replicated using Column A of each Supplemental Table.

## Figures and Tables

**Table 1:** Cross reference with the literature of the 20 most abundant proteins identified in this work.

Tomato Protein Name	This Work		Prior Work		
	Accsn	Ab.	Name	Accession	Primary Reference
Sieve Element Occlusion (P-protein)	Solyc03g111820	781	SEO-F1	A8C977 (1)	Knoblauch et al. 2001 [23]
Bet V 1 protein	Solyc04g005695	669	MLP31	AT5G28010.1 (2)	Carella et al. 2016 [8]
Ribulose bisphosphate carboxylase large chain	A0A0C5CHE6	314	RbcL	ATCG00490.1 (2)	Rodriguez-Celma et al. 2016 [15]
Acyl-CoA Binding domain-containing protein	Solyc08g075690	289	ACBP6	AT1G31812 (2)	Ye et al. 2016 [20]
Lipoxygenase LoxC	Q96573	253	LoxC	Q96573 (1)	Hause et al. 2003 [21]
Stress-response A/B barrel domain-containing protein	Solyc11g066950	234	N/A	AT5G22580 (2)	Z Rahmat 2012 (PhD Thesis) [22]
Glyceraldehyde-3-phosphate dehydrogenase	Solyc05g014470	223	GAPC-2	AT1G13440 (2)	Batailler et al. 2012 [13]
Bet V 1 protein (likely Polyketide cyclase/dehydrase, lipid transport protein, or MLP)	Solyc10g048030	215	MLP43	AT1G70890	Giavalisco et al. 2006 [25]
Peroxidase	Solyc01g006300	197	N/A	XM_023130287 (3)	Walz et al. 2002 [26]
Nucleoside diphosphate kinase	Solyc01g089970	174	NDPK1	AT4G09320 (2)	Batailler et al. 2012 [13]
Malate dehydrogenase	Solyc09g090140	171	MDH	Q7XDC8 (1)	Du et al. 2015 [27]
Nascent polypeptide-associated subunit β	Solyc07g008720	169	BTF3	AT1G17880 (2)	Lin et al. 2009 [7]
Peptidyl-prolyl cis-trans isomerase (PPIase, Cyclophilin)	Solyc01g111170	168	CYP3	AT2G16600 (2)	Deeken et al. 2008 [28]
Uncharacterized protein	Solyc05g054760	159	DHAR2	AT1G75270 (2)	Walz et al. 2002, 2004 [26,29]
Histone H4	Solyc11g072840	156	HIS4	AT2G28740 (2)	
Bet_v_1 domain-containing protein	Solyc04g007820	145	MLP43	AT1G70890 (2)	Giavalisco et al. 2006 [25]
Uncharacterized protein	Solyc07g045440	140	FLA2	At4G12730 (2)	Anstead et al. 2013 [30]
SCP domain-containing protein (likely PR-protein)	Solyc02g065470	135	CAP	At5G66590 (2)	
Non-specific lipid-transfer protein 2 (LTP 2)	Solyc10g075090	131	LTP2	AT2G38530 (2)	
Acidic 27 kDa endochitinase (likely PR protein)	Solyc02g082930	131	CHIB/PR3	AT3G12500 (2)	Rodriguez-Celma et al. 2016 [15]

Abbreviations: 1 = UniProt; 2 = TAIR; 3 = NCBI; Ab., Abundance; Accsn, Accession.

## Supplementary Materials

Supplementary materials can be found at https://www.mdpi.com/1422-0067/21/12/4461/s1. **Table S1:** Main data analysis table containing protein IDs and normalized abundance estimations; **Table S2:** Total phloem exudate protein profile GO term enrichment analysis output from Panther and REVIGO; **Tables S3–S7:** GO term enrichment analysis output from Panther using proteins from hierarchical clustering analysis clusters C1 to C5.

## Abbreviations

ANOVA	Analysis of Variance
BCAA	Branched-Chain Amino Acid
BP	Biological Process
DPS	Days Post Sowing (of seeds)
EDTA	Ethylenediaminetetraacetic Acid
FDR	False Discovery Rate
GO	Gene Ontology
HCA	Hierarchical Cluster Analysis
PCA	Principal Component Analysis
PCC	Pearson Correlation Coefficient
ROS	Reactive Oxygen Species
